# Geochemical fractionation and environmental risk assessment of potentially toxic elements (Cu, Zn, Pb, Ni, Cr, Ba, Mn) in Tokaj vineyard soils, Hungary

**DOI:** 10.1007/s10653-026-03198-5

**Published:** 2026-04-21

**Authors:** Nhung Thi Ha Pham, Ivan Kushnov, Andrea Farsang, Béla Raucsik, Zalán Tobak, Gábor Kovács, Izabella Babcsányi

**Affiliations:** 1 Faculty of Environmental Sciences, University of Science, Vietnam National University, Hanoi, 334 Nguyen Trai, Thanh Xuan, Hanoi, Vietnam; 2 Department of Applied Ecology, Faculty of Biology, St. Petersburg State University, 7/9 Universitetskaya Nab, St. Petersburg, 199034 Russia; 3 Department of Physical and Environmental Geography, University of Szeged, Egyetem U. 2-6., Szeged, 6722 Hungary; 4 Department of Geology, University of Szeged, Egyetem U. 2-6., Szeged, 6722 Hungary; 5 Department of Atmospheric and Geospatial Data Sciences, University of Szeged, Egyetem U. 2-6., Szeged, 6722 Hungary; 6 Department of Horticulture, Faculty of Technical and Human Sciences, Sapientia Hungarian University of Transylvania, Calea Sighișoarei 2, Târgu Mureș/Corunca, 540384 Romania

**Keywords:** Copper, Zinc, Lead, Heavy metals, Sequential extraction, Winegrowing, Agriculture, Erosion

## Abstract

In our study, the sequential extractions and environmental risk assessment of Cu, Zn, Pb, Ni, Cr, Ba, Mn were performed in soil depth profiles and in sediments transported by surface runoff in two vineyards with contrasted soil pH in NE Hungary. Our data indicate that while both sites showed Cu contamination from fungicide applications, the slightly acidic soil in Tállya had considerably higher Cu levels (131 ± 38 mg/kg) than the alkaline soil in Tokaj (51 ± 15 mg/kg) due to longer-term pesticide use history in the former. Potentially toxic elements (PTEs) exhibited overall low mobility in both vineyard soils, with Cr and Ni being strongly retained in the residual fractions (≥ 64–95%). However, Cu revealed high extractability in Tállya (> 57%) down to a depth of 40 cm, further corroborating its predominantly anthropogenic origin and more labile character within the slightly acidic conditions. Contamination and risk assessments using the geoaccumulation index (I_geo_) and the Risk Assessment Code (RAC) showed similar patterns between the two vineyards: while Ni, Cr, Mn, Ba, and Pb were of geogenic origin, Cu and Zn exhibited moderate to heavy contamination status (I_geo_ up to 3.17 (sediment)-3.34 (topsoil) for Cu in Tállya), with sediments showing Cu enrichment compared to topsoil in Tokaj. Copper emerged as the dominant concern, reaching medium risk levels (RAC ≥ 10%) at both sites due to substantial proportions detected in the acid-soluble fraction. This study highlights two critical management priorities for winegrowers: monitoring mobile PTE fractions and preventing contaminated sediment transport to adjacent surface waters.

## Introduction

The accumulation of potentially toxic elements (PTEs) in agricultural soils, particularly in vineyard soils, is a significant environmental issue in historical wine-growing regions worldwide (He et al., 2004; Islam et al., 2015; Mirzaei et al., 2020). The PTE enrichment of vineyard soils is mainly due to the use of metal-based plant protection products (Brunetto et al., 2016; Milićević et al., 2018; Mirzaei et al., 2020). In Europe, copper-based fungicides have been used intensively to protect grapevine against fungal diseases since the late nineteenth century. This practice led to high levels of Cu accumulation in vineyard soil and surrounding areas (Huzum et al., 2012; Milićević et al., 2018; Patinha et al., 2018). Foliar fertilization with micronutrient-rich fertilizers containing Fe, Mn, Cu, Zn, Mo, and B is also part of viticultural practices that may further increase PTE contents in vineyard soils (Pham et al., 2022a). The use of biosolids can also introduce additional PTE contents into agricultural soils, including Pb, Ni, Zn, Cu, Cr, and even some rarely tested metals such as Ba (Ippolito & Barbarick, 2006; Torri & Corrêa, 2012). High levels of PTEs in eroded sediments that accumulate in vineyard toeslope areas can be transported by runoff to streams and canals in valleys, thereby presenting ecological risk to aquatic ecosystems (Martínez-Casasnovas & Ramos, 2006; Pham et al., 2022b; Quinton & Catt, 2007).

Some previous studies have found high contents of PTEs in eroded sediments, indicating a potential ecological risk to aquatic environments (streams, lakes, wetlands) downstream of vineyards (El Azzi et al., 2013; Fernández-Calviño et al., 2012; Pham et al., 2022b). The aquatic ecosystems of urban (Babcsányi et al., 2019) and agricultural areas (El Azzi et al., 2013) are particularly vulnerable, as they act as sinks for PTEs from various human activities and are often in a degraded status. On the other hand, in both alkaline and acidic soils, PTEs can move downward within the soil column, leading to a significant redistribution of their labile fractions towards the subsoil (Blotevogel et al., 2017; Ferreira et al., 2024; Sonoda et al., 2019). A potential risk of plant toxicity may arise from this redistribution process, especially in the rhizosphere zone. In soils and sediments, PTEs can occur in various chemical forms, which largely determine their lability and bioavailability (Arenas-Lago et al., 2014; Fernández-Calviño et al., 2012; Rinklebe & Shaheen, 2014). The chemical forms of PTEs are strongly influenced by soil properties and composition, particularly by soil pH, content and nature of soil organic matter, percentage of silt- and clay-sized fractions, the presence of Fe and Mn oxides/hydroxides, and clay minerals (Arenas-Lago et al., 2014; Bacon & Davidson, 2008). The geochemical fractionation of soil-bound PTEs is commonly assessed by sequential chemical extractions, which can provide a more realistic assessment of their environmental risks than total element analysis.

 The sequential extraction method developed within the Standards, Measurements and Testing Program (SM&T, formerly BCR) of the European Commission has been widely used in metal fractionation studies for soils and sediments. However, the original extraction method suffered from poor reproducibility (Rauret et al., 1999). The improved BCR (thereafter referred to as SM&T), a three-step sequential extraction method, provided a simplified procedure for evaluating the geochemical forms of soil- and sediment-bound PTEs, and it has since been widely used (Nemati et al., 2011; Soliman et al., 2018; Tong et al., 2020). The SM&T method can distinguish between acid-soluble fraction (exchangeable and carbonate-bound), elements bound to Mn and Fe oxides and hydroxides (reducible fraction), elements associated with organic matter (oxidizable fraction), and elements in the residual fraction (incorporated into the crystal structure of minerals) (Kazi et al., 2006). The acid-soluble fraction has been used in environmental risk assessment studies as the at-risk PTE fraction, as it represents the most labile form of soil-bound PTEs and is considered the bioavailable fraction in some studies (Li et al., 2018; Nemati et al., 2011; Nkinahamira et al., 2019). Accordingly, the risk assessment code (RAC), which considers the proportion of the acid-soluble fraction in relation to the sum of all extracted fractions of PTEs, has been commonly applied to determine the environmental risk of PTE contaminants (Li et al., 2018; Tong et al., 2020; Wieczorek et al., 2023). In our study, environmental risk of PTEs in the soils and eroded sediments was calculated based on the PTE contents recovered in the first step of the SM&T extraction method, using the RAC method. 

This study aims to assess the degree of contamination and environmental risk of selected PTEs in soils and eroded sediments based on the geoaccumulation index (I_geo_) and the geochemical fractions in two sloping Hungarian vineyards with contrasted soil properties in terms of pH. Both vineyards are located near permanent or temporary streams and stormwater ponds, necessitating evaluation of the environmental risks posed by the transport of soil-bound PTEs. Environmental risk assessment is important to inform winegrowers and encourage implementation of erosion-control measures. In line with previous works on vineyard soils (Milićević et al., 2018; Mirzaei et al., 2020; Ribolzi et al., 2002), this study focuses on some risk elements, namely Cu, Zn, Pb, Ni, Cr as common soil contaminants, and other, less frequently studied PTEs, such as Mn and Ba. The specific objectives of our research were to: (1) determine the geochemical fractionation of the target PTEs (Cu, Zn, Pb, Ni, Cr, Ba, Mn) in soils and eroded sediments from two historical Hungarian vineyards displaying contrasted pH (slightly alkaline and slightly acidic); (2) assess the environmental risk of these PTEs by applying the risk assessment code (RAC), and calculating the geoaccumulation index. The primary novelty of this work lies in the integrated methodological approach that combines sequential extraction (SM&T), correlation analysis, and risk assessment (I_geo_, RAC) to characterize PTE geochemical behavior, lability and environmental risks in soil depth profiles, topsoils, and eroded sediments from two vineyards with contrasting soil pH within the same wine-growing region.

## Materials and methods

### Study area and sampling scheme

**Fig. 1:**
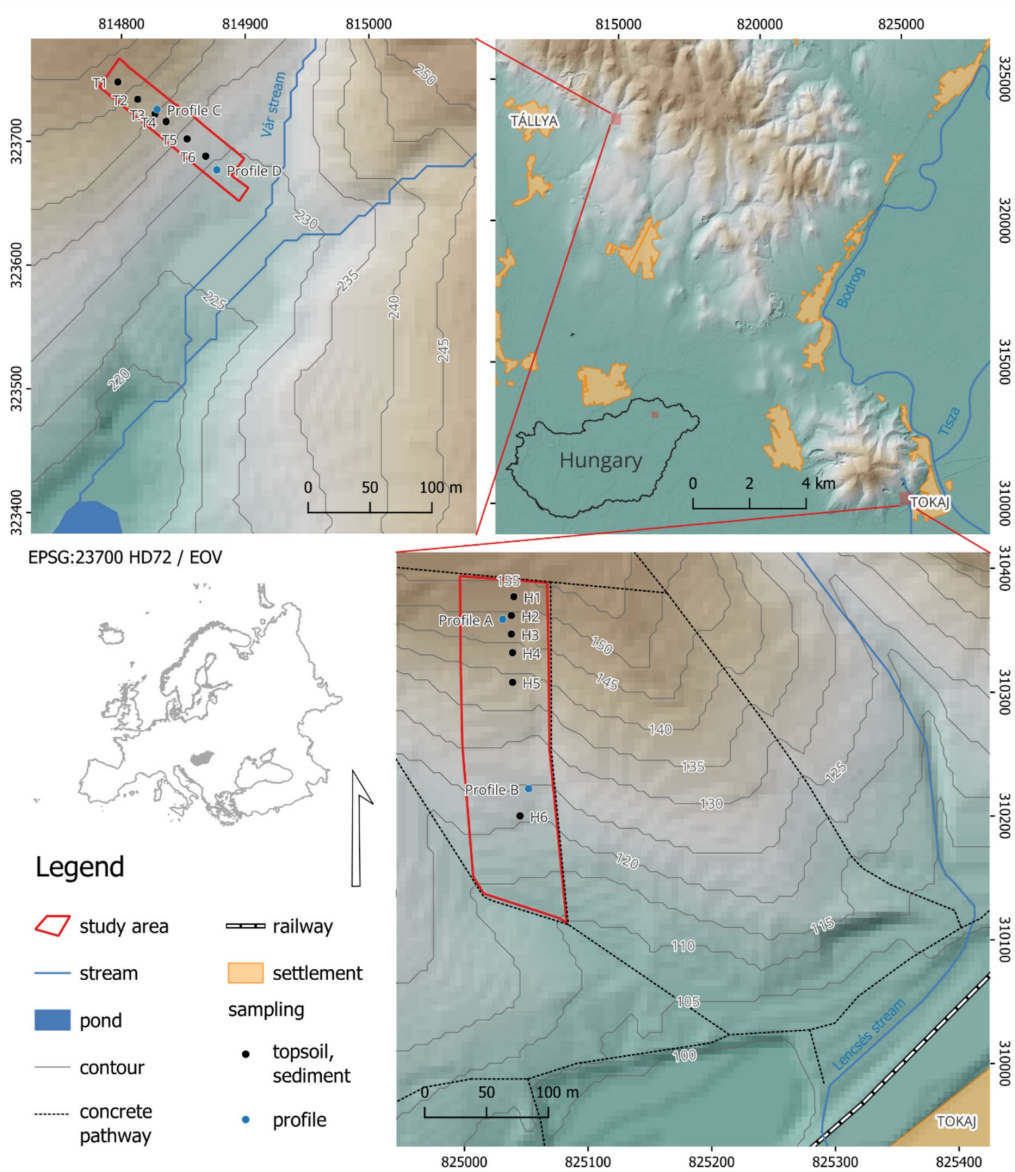
Location of the two vineyards under study, Tállya and Tokaj, in the Tokaj-Hegyalja wine region (North-Eastern Hungary). The locations of soil profile sampling points (profiles A, B, C, and D), sediment traps, and topsoil sampling points are also shown

The study was conducted in a 1.8 ha vineyard plot in Tokaj (mean slope of 8°, slope length 270 m) and a 0.4 ha plot in Tállya. In Tállya, the mean slope of the plot is 18°, and the total slope length is 146 m, with a marked inflection point at the midpoint of the slope section. Both vineyards are located in the Tokaj-Hegyalja wine region, NE Hungary (Fig. 1). In Tállya, the study area is part of the Halastó vineyard and is less than 10 m from the Vár Stream, a small temporary stream that transports water and sediments to a rainwater storage pond located 270 m from the studied vineyard. The vineyard plot in Tokaj is situated in the Hétszőlő vineyards, 250 m from the temporary Lencsés stream, which gathers surface runoff from both the Hétszőlő and Lencsés vineyards. A network of concrete field paths was constructed in the Hétszőlő vineyard to capture and channel surface runoff into the Lencsés stream, which effectively serves as a stormwater drainage system (Fig. 1) that periodically discharges into irrigation channels connected to the Tisza River.

The soil in Tállya has diverse characteristics along the slope profile. According to the World Reference Base for Soil Resources (2015), a Skeletic Regosol (Loamic, Ochric) type is found at the top of the hillslope, while at the backslope, the vineyard soil turns into a Skeletic Leptosol (Loamic, Ochric). At the footslope area, the soil is classified as Skeletic Colluvic Regosol (Loamic, Ochric). The rhyolite tuff and andesite base rocks constitute the soil-forming parent material in the vineyards of Tállya (Zelenka et al., 2012). The beginning of grape growing in this region supposedly dates back more than 100 years. In the year of the study (2019–2020), the Bordeaux mixture complemented with a foliar micronutrient fertilizer (Fe (3.2 m/V%), Mn (0.32 m/V%), Cu (0.15 m/V%), B (0.31 m/V%) and Mo (0.003 m/V%)) was pulverized three times in a dose of 4–5 l/ha delivering a total of 0.5–0.6 kg/ha Cu in the vineyard.

The primary parent material in Tokaj is loess that covers the late Miocene stratovolcano, built up of pyroxene dacite and pyroxene dacite tuffs (Harangi & Lenkey, 2007; Novák et al., 2014; Zelenka et al., 2004). The vineyard soil is a Calcaric Regosol (Siltic, Ochric). The soil is weakly developed due to intense erosion, preventing the formation of diagnostic horizons. Before the 1993 replanting, the area had not been under vine since the 1950s. Before the 1950s, however, the area was cultivated as a historic vineyard for a long time. The study plot has been managed in accordance with organic farming guidelines since 1993. Fresh cattle manure of 0.3 t/ha was regularly used to fertilize vineyards every 3–4 years, but in the last 10 years, farmers have been using cattle manure pellets.

Copper-fungicide applications have taken place over the past 26 years with a typical dose of 4 kg/ha/year (metallic Cu), supplemented with sulfur-containing pesticides and foliar fertilizers containing macro- and micronutrients (K_2_O, N_org_, C_org_, MgO, S, B, Zn, Mn, Mo, Cu, Fe). However, no data are available on vine cultivation practices (including the possible use of Cu) before the 1950s. In Europe, the use of Cu fungicides began in the late nineteenth century, and their widespread use dates back to the end of World War II (Sabatier et al., 2014). Therefore, it is assumed that most of the pesticide-derived Cu in the Hétszőlő vineyard has been applied since the vineyard was replanted in 1993.

The soil samples were collected in March 2019 from four soil profiles (two per vineyard) to a depth of 2 m (Fig. 1). The following soil layers were sampled: 0–10 cm; 10–20 cm; 20–30 cm; 30–40 cm; 60–80 cm; 120–140 cm; 180–200 cm. At the same time, Gerlach-type sediment traps were placed along the main slope transect at each sampling location (Fig. 1). To compare the quality of the sediment and the topsoil, the 0–20 cm topsoil layer was also collected near each trap. In May, 12 sediment samples were collected from the traps following rainfall events. Eight distinct rainfall events occurred during the sediment sampling period (14.05.2019 to 19.05.2019) in Tállya, with an average rainfall intensity of 0.26 mm/h, a maximum intensity of 1.60 mm/h, and a total rainfall depth of 4 mm. In Tokaj, 6 rainfall events were recorded with a mean rainfall intensity of 1.55 mm/h, a maximum intensity of 5.50 mm/h, and a total depth of 16 mm during the same sampling period. Rainfall data were continuously recorded on-site using a rain gauge (Type BCU LITE2, Boeras Ltd., Hungary).

### Soil characterization and sequential extraction methods

The collected soil samples were air-dried, and sediment samples were oven-dried overnight at 80 °C. All samples were disaggregated in a mortar and then sieved to pass through a 2-mm sieve. The hygroscopic moisture content of the prepared soil samples was determined by oven-drying at 105 °C for 24h. The soil pH (in deionized water) was determined in a soil: deionized water (1:2.5) mixture using a digital pH meter (Inolab pH 720) (± 0.05). The organic matter content was analyzed by a UV–VIS spectrophotometer (a type Spectronic Helios-γ, Thermo Fisher Scientific), following H_2_SO_4_-aided oxidation of the organic matter with 0.33 M K_2_Cr_2_O_7_ (with an uncertainty of ± 2%). The carbonate content was measured using the Scheibler method (uncertainty ± 8%) (Bojar et al., 2020). The pipette method was used to determine the particle size distribution of the soil samples after dispersing them in a 0.1 M solution of sodium pyrophosphate, as specified in the Hungarian standard MSZ-08-0205 (1978). Soil texture was classified according to the United States Department of Agriculture (USDA) classification system (Moreno-Maroto & Alonso-Azcárate, 2022). A Rigaku Ultima IV diffractometer was employed for XRPD measurements, configured with Bragg-Brentano geometry, CuKα radiation, single-crystal graphite monochromator, proportional counter detector, and both divergence and detector slits set at 2/3°. For whole-sample analysis, random powder mounts were prepared using ~ 0.04 g of soil powder on a Si single-crystal sample holder to determine the mineralogical composition. Specimens were scanned at 50 kV/40 mA from 3 to 70°2θ with a goniometer step rate of 1°/min and data acquisition steps of 0.05°. The qualitative evaluation of the XRPD diffractograms was performed with the Rigaku PDXL 1.8 software, using the ICDD (PDF2010) database. A semi-quantitative mineralogical composition was estimated using the reference intensity ratio (RIR) method.

Selected soil and sediment samples (3 topsoils, 3 sediment samples/site, and some soil profile layers, listed above) were subjected to sequential extraction according to the improved three-step plus a residual digestion step SM&T method proposed by Rauret et al. (1999). Beforehand, the samples were finely ground in an agate ball mill to pass through a 250 µm sieve. Briefly, the SM&T method consists of extracting the acid-soluble and easily mobilizable (mainly exchangeable and carbonate-bound) PTEs with acetic acid (0.11 mol/l) (F1), in the second step (F2: reducible fraction) the PTEs bound to Fe and Mn oxyhydroxides with a freshly prepared hydroxylamine hydrochloride solution (0.5 mol/l), in the third step (F3: oxidizable fraction) the PTEs bound to organic matter and sulfides by treating the samples with hydrogen peroxide (8.8 mol/l) and ammonium acetate (1 mol/l). In the fourth step (F4: residual fraction), the PTEs within the crystal structure of minerals were dissolved in aqua regia. For the last step, 0.5 g of dried sample material (recovered after step F3) was weighed into a PFA vessel, and 7 mL of aqua regia (nitric acid: hydrochloric acid = 1:3) was added for microwave-assisted digestion (Anton Paar Multiwave 3000). The chemical element concentrations in the extracts were determined by an inductively coupled plasma optical emission spectrometer (ICP-OES) (Optima 7000 DV, PerkinElmer). Some samples have been treated in duplicate to account for the reproducibility of the sequential extraction procedure, which has an uncertainty of ≤ 20%. For quality control purposes, four aliquots of a certified reference soil material (ERM®-CC141, a loam soil) were digested the same way as the residual fraction in aqua regia and analyzed by ICP-OES in six different measurement series (2 digested aliquots were measured twice, and two aliquots once). The measured element contents were compared with the certified values, and the following recoveries were obtained: 106 ± 3% (Cu), 97 ± 8% (Zn), 92 ± 8% (Pb), 120 ± 2% (Ni), 119 ± 2% (Cr), and 104 ± 2% (Mn). The reference soil was not certified for Ba. The reproducibility of the elemental content measurements by ICP-OES was better than 10%. We acknowledge a systematic bias (+ 20%) in Ni and Cr recoveries that highlight a possible slight overestimation of the residual Ni and Cr contents.

### Environmental risk assessment of PTEs

In the present study, well-established quantitative indices, such as geoaccumulation index (Igeo) and risk assessment code (RAC), commonly used in soil studies (particularly for PTE geochemical fractions and risk assessment), were employed to evaluate potential pollution and environmental risk from PTEs (Li et al., 2018; Tong et al., 2020; Wieczorek et al., 2023). These indices were calculated based on the contents of SM&T extracted fractions of PTEs in studied soils and sediments.

The geoaccumulation index (I_geo_) originally suggested by Müller (1979) was applied here to assess the degree of PTE accumulation by comparing the contents of the PTE in the topsoil or sediment with its background content in an uncontaminated reference material (the deepest subsoil layer in our study). It was expressed as follows:

$$\text{Igeo }={\mathit{log}}_{2}(\frac{{\mathrm{C}}_{\mathrm{n}}}{{1.5\text{ x B}}_{n}})$$

where C_n_ and B_n_ are the soil/sediment-bound overall contents of a given PTE recovered in four SM&T extracted fractions in the topsoil/sediments and in the subsoil (180–200 cm) chosen as the reference soil. In Tállya, the deepest layer in profile D was used, while in Tokaj-Hétszőlő the average of the two deepest layers was used in the calculations. The factor 1.5 was used considering the variation of background values due to regional differences. The I_geo_ is classified into 7 levels of contamination (Müller, 1979): I_geo_ ≤ 0 (uncontaminated), 0 < I_geo_ ≤ 1 (uncontaminated to moderately contaminated), 1 < I_geo_ ≤ 2 (moderately contaminated), 2 < I_geo_ ≤ 3 (moderately to heavily contaminated), 3 < I_geo_ ≤ 4 (heavily contaminated), 4 < I_geo_ ≤ 5 (heavily contaminated to extremely contaminated) and I_geo_ > 5 (extremely contaminated).

The risk assessment code (RAC) is also a useful indicator of environmental risk of each PTE based on its chemical forms (Wieczorek et al., 2023). The RAC represents the percentage of the exchangeable/acid-soluble PTE contents, accordingly, it is calculated as follows:

$$RAC=\left(\frac{F1}{F1+ F2 + F3+F4}\right)x 100\%$$

The classification of the RAC is described in 5 levels: safe level (less than 1%), low risk level (1–10%), medium risk level (10–30%), high risk level (30–50%) and very high risk level (over 50%).

### Data analysis

Descriptive statistical analysis and data processing of the studied soil and sediment characteristics were carried out using IBM SPSS software (version 25). Spearman rank-order correlation analysis was performed to determine the relationships between PTE data and soil properties. The significance level was considered at p < *0.05* and p < *0.01*. In addition, one-way ANOVA was used to examine differences among the means of the soil properties in the top- and subsoil layers at the level of p < *0.05.*

## Results and discussion

### Soil properties and PTE soil contents in the studied vineyards

The vineyards of Tállya and Tokaj-Hétszőlő exhibit distinct soil characteristics in terms of pH, carbonate levels, and particle-size distribution (Table 1). Tállya’s slightly acidic soils, formed over rhyolite tuff and andesite bedrock, contain minimal SOM and carbonate. In contrast, Tokaj-Hétszőlő’s moderately alkaline soils have medium carbonate levels but similarly low SOM contents (< 2%). Carbonate contents are evenly distributed within the soil profiles A and B of Tokaj-Hétszőlő, attributed to the presence of dolomite and calcite minerals in the loess material that forms the soil (Table 1). XRPD analysis revealed that topsoils contain only trace amounts of these carbonate phases, with quartz being the dominant mineral (60–70%) and 10–20% of 10 Å phases (illite/muscovite) (Table 2). The soil also comprises 5–10% of 14 Å phases (chlorite/vermiculite) and 5–10% albite. In the soils from Tállya, XRPD results confirm minimal carbonate content, with calcite and dolomite just above the detection limit. Major constituents include quartz (50–60%), potassic feldspars (~ 10%), and albite (~ 10%), while 10 Å clay minerals (illite/muscovite) occur only in trace amounts. The low SOM content at both sites likely results from intensive soil erosion and removal of organic-rich particles. The mobilization of organic-rich particles by soil erosion is supported by the collected sediments containing higher organic matter (averaging 2.8–3.1%) than topsoils. Such low SOM levels (< 2%) are typical of intensively cultivated vineyards on steep terrain. In Tállya, erosion is exacerbated by inter-row tillage and vine rows planted parallel to the slope (Novara et al., 2018). Consequently, profile C in Tállya represents the shallowest soil (≤ 120 cm), while profile D at the toeslope exhibit ts effects of re-sedimentation, as the soil cover extends beyond 200 cm depth. Textural variation along the main slope in Tállya is mainly caused by particle-size sorting during water erosion and transport processes. Profile C’s clay loam texture transitions to sandy loam in profile D at the toeslope. In contrast, the silt loam soils of Tokaj-Hétszőlő show no notable textural differences between sampling sites A and B. In Tállya, sediment mineralogy varied by location: T3 (near the inflection point) contained more quartz (60–70% vs. 50–60% in topsoils) with similar illite/muscovite content (5–10%), while T5 (near the toeslope) showed quartz levels comparable to topsoils but enrichment in illite/muscovite (10–20%). These differences may reflect particle-size sorting during downslope transport, with coarser fractions dominating at T3 and finer clay minerals in T5. Nevertheless, the mineralogical composition of sediments in Tokaj reveals elevated proportions of 10 Å illite/muscovite (20–30% in H2 and 40–50% in H5), compared to topsoil (10–20%), indicating enrichment of clay minerals in transported soil material (Table 2).

**Table 1:** Characteristics of the topsoil, soil profiles, and eroded sediments in the two vineyards studied, Tállya and Tokaj-Hétszőlő

Soil depth (cm)	SOM* (%)	pH	CaCO_3_ (%)	Clay %	Silt %	Sand %	Soil texture +
Tokaj topsoil (n = 6)	1.99 ± 0.36	8.16 ± 0.07	4.31 ± 0.74	16.94 ± 1.44	72.15 ± 2.28	10.91 ± 2.88	Silt loam
Tokaj sediment (n = 6)**	3.14 ± 0.23	–	4.93 ± 0.72	–	–	–	–
Tokaj–profile A							
0–10	1.70	7.96	4.19	19.65	58.16	22.19	Silt loam
10–20	1.79	7.94	4.19	10.75	63.30	25.95	Silt loam
20–30	1.20	8.01	4.60	19.07	68.10	12.83	Silt loam
30–40	0.96	8.01	3.77	25.55	62.22	12.22	Silt loam
60–80	0.57	8.38	5.41	23.20	68.98	7.82	Silt loam
120–140	0.37	8.52	6.24	20.10	72.34	7.57	Silt loam
180–200	0.51	8.21	5.44	14.43	74.61	10.96	Silt loam
Tokaj–profile B							
0–10	0.72	8.23	4.48	15.62	68.62	15.76	Silt loam
10–20	1.03	8.24	4.99	18.27	68.19	13.54	Silt loam
20–30	0.67	8.37	3.74	18.53	68.34	13.14	Silt loam
30–40	0.48	8.36	4.16	19.23	67.02	13.74	Silt loam
60–80	1.41	8.23	4.57	15.31	59.84	24.85	Silt loam
120–140	1.35	7.98	4.57	15.63	65.42	18.94	Silt loam
180–200	1.32	7.85	3.77	23.36	68.64	8.00	Silt loam
Tállya topsoil (n = 6)	1.63 ± 0.43	6.38 ± 0.47	1.89 ± 0.12	35.61 ± 6.71	29.10 ± 4.01	35.29 ± 9.26	Clay loam
Tállya sediment (n = 6)**	2.79 ± 0.70	–	1.60 ± 0.19	–	–	–	–
Tállya–profile C							
0–10	1.26	6.18	2.94	33.57	33.57	32.86	Clay loam
10–20	1.11	6.16	2.94	–	–	–	–
20–30	0.66	6.22	2.49	41.78	23.87	34.35	Clay
30–40	0.50	6.13	2.07	–	–	–	–
60–80	0.30	5.11	1.66	41.18	24.32	34.50	Clay
120–130	0.83	6.28	3.36	17.39	22.19	60.42	Sandy Loam
Tállya–profile D							
0–10	1.63	6.63	2.94	12.59	24.86	62.55	Sandy loam
10–20	1.02	6.45	3.78	–	–	–	–
20–30	0.75	6.28	1.66	19.33	25.86	54.82	Sandy loam
30–40	0.55	6.42	1.66	–	–	–	–
60–80	0.58	6.80	2.07	19.52	24.62	55.86	Sandy loam
120–140	0.37	6.80	2.07	25.39	27.71	46.90	Loam
180–200	0.66	6.58	2.10	23.26	32.16	44.58	Loam

*SOM: Soil organic matter content; + Classification of soil texture according to USDA system. **Due to insufficient sample quantities, pH and grain size distribution of sediments could not be determined

**Table 2:** Mineralogical composition of the soils and sediments in the studied vineyards

	Major constituents	Minor constituents
Profile A (0–10 cm)	quartz (60–70%), 10 Å (10–20%)	14 Å (5–10%), albite (5–10%), dolomite (traces), calcite (traces), goethite? (traces), amphibole? (traces), smectite? (traces)
Profile B (0–10 cm)	quartz (60–70%), 10 Å (10–20%)	14 Å (5–10%), albite (5–10%), dolomite (traces), calcite (traces), smectite? (traces)
H2 sediment	quartz (~ 50%), 10 Å (20–30%)	14 Å (5–10%), albite (5–10%), dolomite (traces), calcite (traces)
H5 sediment	quartz (30–40%), 10 Å (40–50%)	14 Å (~ 10%), albite (traces), dolomite (traces), calcite (traces), K-feldspars (traces)
T1 soil	quartz (50–60%), K-feldspars (~ 30%)	10 Å (5–10%), 14 Å (traces), albite (traces), smectite (traces), dolomite? (traces), calcite? (traces), 7 Å? (traces)
T4 soil	quartz (50–60%), K-feldspars (~ 10%), albite (~ 10%)	10 Å (traces), smectite (traces), dolomite? (traces), calcite? (traces), 7 Å? (traces)
Profile D (0–10 cm)	quartz (50–60%), K-feldspars (~ 10%), albite (~ 10%)	10 Å (traces), smectite (traces), dolomite? (traces), calcite? (traces)
T3 sediment	quartz (60–70%), albite (~ 10%)	10 Å (5–10%), 7 Å (traces), K-feldspars (traces), smectite (traces)
T5 sediment	quartz (50–60%), 10 Å (10–20%)	K-feldspars (5–10%), albite (5–10%), 7 Å (traces), smectite (traces)

**Fig. 2:**
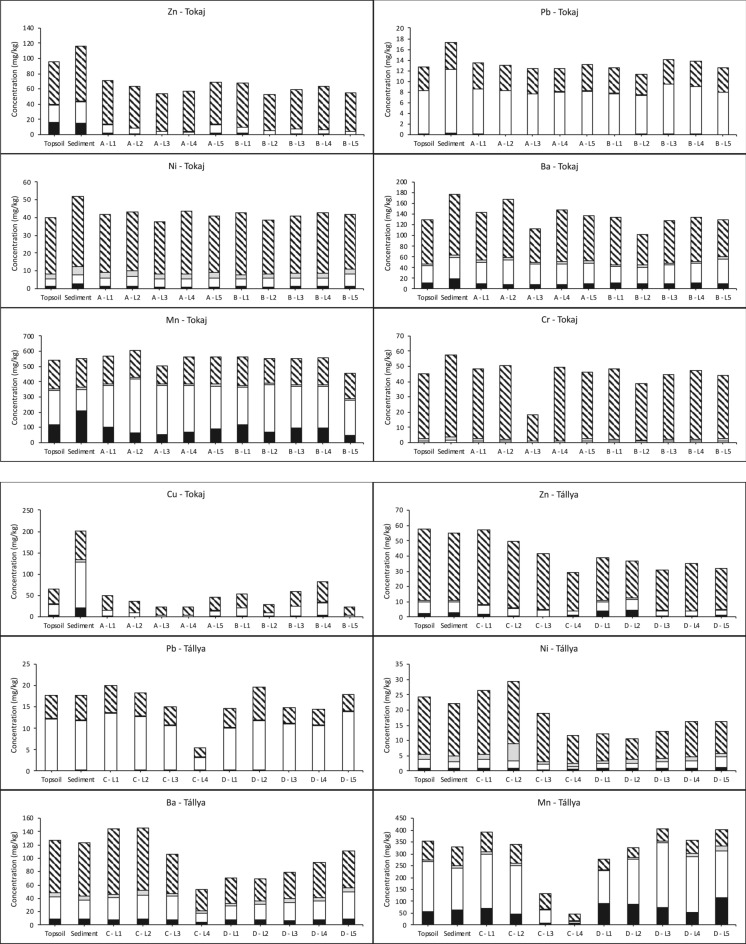

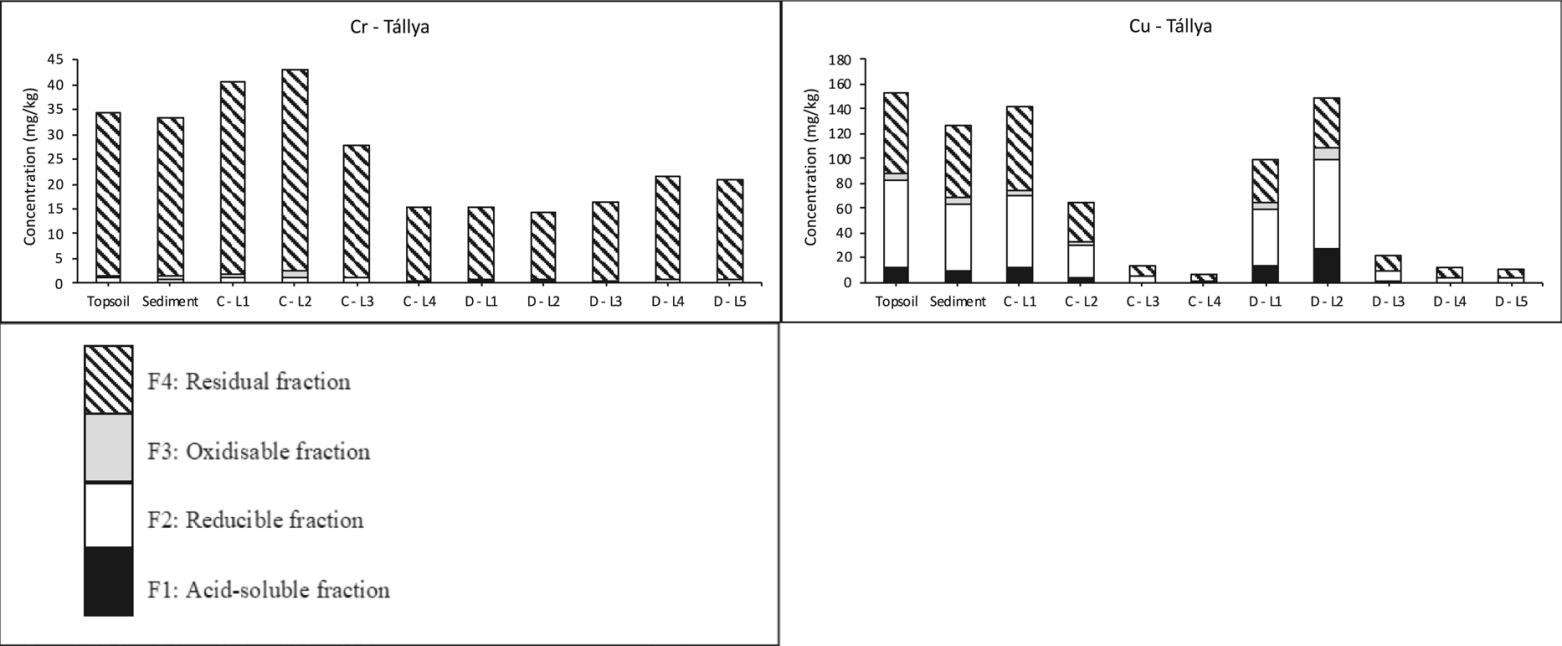
Extracted PTE contents (mg/kg) in soils and sediments collected from the Tokaj-Hétszőlő and Tállya vineyards. The soil layers are labelled as follows: L1: 0–20 cm soil layer; L2: 20–40 cm soil layer; L3: 60–80 cm soil layer; L4: 120–140 cm soil layer; L5: 180–200 cm soil layer

PTE contents (Fig. 2) in the studied vineyards generally fall below World Soil Average values for uncontaminated soils (Pb < 27 mg/kg, Cr < 60 mg/kg, Ba < 460 mg/kg), with exceptions for Ni and Zn (Ni > 29 mg/kg and Zn > 70 mg/kg in Tokaj) and Cu (> 39 mg/kg at both sites) (Kabata-Pendias, 2010). Copper data show clear human-induced contamination, along with minor Zn accumulations, in Tokaj topsoil, whereas the slightly elevated Ni levels in Tokaj are more likely to be naturally occurring. Of the two vineyards, Tokaj displays higher PTE levels than the other vineyard because its loess subsoil naturally contains higher contents, reflecting a greater geochemical background. The vertical distribution of Pb (13 ± 1 mg/kg), Ni (41 ± 3 mg/kg), and Mn (547 ± 36 mg/kg) in the profiles A and B in Tokaj show no discernible patterns, indicating no or negligible anthropogenic accumulation (Fig. 2). In Tállya, Mn, Cr, Ba, and Ni display increasing contents with depth in profile D within the sedimentation zone. In contrast, the upslope soil (profile C) exhibits the reverse pattern (Fig. 2). The markedly reduced clay content in profile D’s surface layers (34% in profile C versus 13% in profile D), coupled with correspondingly lower Mn, Cr, Ba, and Ni levels, indicates that erosion predominantly mobilizes coarser (e.g., fine sand) fractions from the hillslopes, which transport fewer trace elements than clays to the foot- and toeslope areas where sedimentation mainly takes place (Manaljav et al., 2021). In Tokaj, standard deviations for Cr and Ba that exceed the 10% measurement uncertainty reflect natural variability from loess deposits with varying elemental compositions and/or element redistribution during loess weathering (Sümegi & Hertelendi, 1998). Variability in the vertical Zn profile (64 ± 8 mg/kg) suggests a modest influence of vineyard management practices on topsoil Zn levels.

Surface layer Cu enrichment was substantially greater in Tállya vineyard (131 ± 38 mg/kg) compared to Tokaj (51 ± 15 mg/kg). Even higher Cu contents have been documented in European and Brazilian vineyards (Nogueirol et al., 2010). Alsatian vineyard topsoils contain Cu ranging from 40 to 188 mg/kg (Duplay et al., 2014), while French Mediterranean vineyards exhibited even broader ranges of 31–250 mg/kg (Brun et al., 2001). Across Europe, vineyard Cu content is thought to vary primarily due to climatic factors (particularly precipitation) and soil organic carbon levels (Droz et al., 2021). Nevertheless, the disparity between Tokaj and Tállya stems mainly from the extended duration of repeated Cu fungicide applications in Tállya. The U-shaped distribution of Cu in profile B at Tokaj indicates substantial contemporary and historical Cu accumulation alongside landscape-modification disturbances. Indeed, elevated Cu contents at depths of 60–80 cm and 120–140 cm may result from Cu accumulation during earlier viticulture, prior to vineyard abandonment in the 1950s. Erosion-sedimentation dynamics and landscape reshaping before the 1993 replanting likely buried former soil surfaces in certain locations.

Sediments in Tokaj displayed substantial Cu enrichment, with contents 2.5 to 3.5 times higher than topsoil values, comparable to findings from Spanish vineyards in Galicia (1.2 to 5.6-fold enrichment) (Fernández-Calviño et al., 2008). By contrast, sediments collected in Tállya showed no PTE enrichment relative to topsoil (Fig. 2). This divergence between sites likely reflects differences in soil texture and composition. The absence of PTE enrichment, including Cu, in Tállya’s eroded sediments aligns with observations that the topsoil in the sedimentation zone exhibits lower PTE contents and coarser texture compared to the upslope profile C (Table 1 and Fig. 2). Clay-sized fractions act as the main carriers of PTEs, and their presence in the soil material moved by overland flow largely controls the downhill transport of these elements. Additionally, previous studies have shown that particle size distribution in the transported sediments can also vary with precipitation patterns (Babcsányi et al., 2016; Imfeld et al., 2020). The comparatively low rainfall energy recorded at Tállya during the sediment collection period (total depth: 4 mm; maximum intensity: 1.60 mm/h; vs. Tokaj: 16 mm, 5.50 mm/h) may have resulted in the preferential transport of coarser sediment fractions characterized by lower PTE concentrations.

### The geochemical fractionation and environmental risk assessment of PTEs in the soil and eroded sediments

**Fig. 3:**
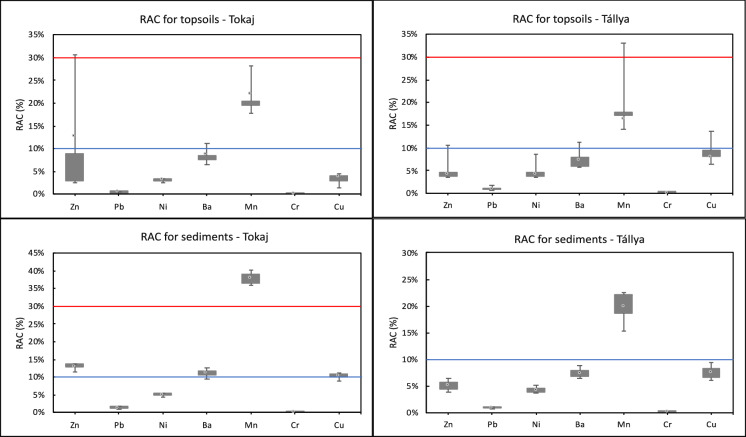
Risk assessment code (RAC) (%) of target PTEs in the vineyard topsoils and eroded sediments in Tállya and Tokaj. The blue line indicates RAC = 10%, above which medium risk level is identified, and the red line stands for RAC = 30%, above which high risk level is identified

Among the PTEs examined, Cr exhibited the highest proportions recovered in the residual fraction, as determined by the sequential extractions (95% in both Tokaj and Tállya) (Fig. 2). Given the absence of notable Cr enrichment in surface layers supported by low I_geo_ values (Table 4), Cr appears to be naturally occurring in these vineyards. Lead and Cr show RAC values below 1.3% in both vineyards (Fig. 3), generally indicating safe levels and no significant environmental risk. The I_geo_ values below 0 for Pb indicate no anthropogenic Pb contamination in either vineyard. However, Pb exhibited a unique fractionation pattern compared to other PTEs, with the residual fraction not predominating in either vineyard. Instead, the reducible (F2) fraction was dominant in both vineyards (Fig. 2). The strong negative correlations between Pb in the acid-soluble (F1: ρ = − 0.81**), reducible (F2: ρ = − 0.73**), and oxidizable (F3: ρ = − 0.59**) fractions with soil pH and carbonate content (Table 3) indicate that Pb mobility is inversely related to alkalinity. This pH-dependent behavior reflects the tendency of Pb to form (co−)precipitates with hydroxides and also carbonates, effectively immobilizing the soil-bound Pb contents (Cerqueira et al., 2011; Kabala & Singh, 2001). The positive correlations between Pb in the reducible (F2) and oxidizable (F3) fractions and clay content (ρ = 0.52** and 0.48*, respectively) further suggest that Pb is preferentially associated with Fe/Mn oxyhydroxides and organic matter bound to clay minerals and/or present in the clay-sized fraction. In the alkaline soils of Tokaj (pH 8.16 ± 0.07) and the slightly acidic conditions of Tállya (pH 6.38 ± 0.47), these immobilization mechanisms explain the consistently low RAC values for Pb at both sites.

**Table 3:** Spearman’s correlation matrix between soil properties and PTE fractionation data

Fraction	Element	SOM*	pH	CaCO_3_	Clay	Silt	Sand
F1 (Acid-soluble fraction)	Zn	0.73^**^	ns	ns	ns	ns	ns
	Pb	ns	− 0.81^**^	− 0.77^**^	ns	− 0.64^**^	0.65^**^
	Ni	0.66^**^	ns	ns	− 0.48^*^	ns	ns
	Ba	0.47^*^	0.41^*^	0.50^**^	ns	0.55^**^	− 0.42^*^
	Mn	ns	ns	ns	− 0.63^**^	0.39^*^	ns
	Cr	ns	ns	ns	ns	ns	ns
	Cu	0.60^**^	ns	ns	ns	ns	ns
F2 (Reducible fraction)	Zn	0.65^**^	ns	ns	ns	0.51^**^	ns
	Pb	ns	− 0.73^**^	− 0.71^**^	0.52^**^	− 0.45^*^	0.49^*^
	Ni	ns	0.70^**^	0.73^**^	ns	0.65^**^	− 0.69^**^
	Ba	ns	ns	ns	ns	ns	− 0.43^*^
	Mn	ns	0.60^**^	0.58^**^	ns	0.50^**^	− 0.45^*^
	Cr	ns	ns	ns	0.42^*^	ns	− 0.39^*^
	Cu	0.68^**^	ns	ns	ns	ns	ns
F3 (Oxidisable fraction)	Zn	0.43^*^	ns	ns	ns	ns	ns
	Pb	ns	− 0.59^**^	− 0.62^**^	0.48^*^	− 0.62^**^	0.50^**^
	Ni	ns	0.52^**^	0.65^**^	ns	0.55^**^	− 0.60^**^
	Ba	ns	− 0.78^**^	− 0.74^**^	0.62^**^	− 0.55^**^	0.46^*^
	Mn	ns	ns	ns	ns	ns	ns
	Cr	0.53^**^	ns	0.42^*^	ns	0.51^**^	ns
	Cu	0.60^**^	− 0.41^*^	− 0.40^*^	ns	ns	ns
F4 (Residual fraction)	Zn	ns	0.66^**^	0.77^**^	ns	0.75^**^	− 0.70^**^
	Pb	ns	ns	ns	ns	ns	Ns
	Ni	ns	0.72^**^	0.82^**^	ns	0.70^**^	− 0.71^**^
	Ba	ns	ns	0.48^*^	ns	0.53^**^	− 0.56^**^
	Mn	ns	0.66^**^	0.72^**^	− 0.41^*^	0.76^**^	− 0.63^**^
	Cr	ns	0.52^**^	0.69^**^	ns	0.60^**^	− 0.61^**^
	Cu	0.67^**^	ns	ns	ns	ns	ns
Soil properties	SOM	1.00					
	pH	ns	1.00				
	CaCO_3_%	ns	0.84^**^	1.00			
	Clay %	ns	− 0.58^**^	− 0.57^**^	1.00		
	Silt %	ns	0.64^**^	0.65^**^	ns	1.00	
	Sand %	ns	− 0.62^**^	− 0.59^**^	ns	− 0.87^**^	1.00

*SOM: Soil organic matter content, ns: not significant. *Significant at the level of p < 0.05; ^**^Significant at the level of p < *0.01*

Approximately 50% of Mn was present in the reducible fraction (as Mn oxides/oxyhydroxides) at both sites. Soil Mn is considered geogenic, with no indication that vineyard management has caused substantial Mn accumulation that is well supported by the I_geo_ values consistently below 0 (Table 4) in both soil and sediments from both sites. Manganese exhibits complex geochemical behavior due to its multiple oxidation states and occurrence in both crystalline and pseudocrystalline oxide structures, with Mn oxides frequently coprecipitating with Fe oxides (Davidson et al., 2006). The relatively low proportions of organic matter-associated Mn (2% in Tokaj and 3% in Tállya) contrast with the greater acid-soluble Mn fractions (F1: 16% in Tokaj and 19% in Tállya). The acid-soluble fraction may include not only exchangeable and carbonate-bound Mn but also unstable Mn-organic complexes that are readily extracted in F1 (Kabata-Pendias, 2010; Sungur et al., 2019). The positive correlation between Mn in the reducible fraction (F2) and silt content (ρ = 0.50**) indicates that Mn oxides are preferentially associated with fine-textured soil fractions. The strong positive correlation between residual Mn (F4) and carbonate content (ρ = 0.72**) reflects the incorporation of Mn into recalcitrant carbonate mineral structures. Manganese is the most abundant metal in the studied soils, with total SM&T-extracted contents below 600 mg/kg, well within acceptable levels for agricultural soils. Additionally, Mn has no established regulatory limit in agricultural soils due to its essential role as a plant micronutrient and generally low toxicity at environmentally relevant contents (Kabata-Pendias, 2010). Nevertheless, the high RAC values of 38% in eroded sediments and 22% in topsoils from Tokaj indicate considerable mobility and bioavailability of this element, which could pose phytotoxicity risks under acidic or waterlogged conditions that promote Mn reduction and dissolution.

**Table 4:** Geoaccumulation Index (I_geo_) based on the pseudo-total PTE contents (sum of F1, F2, F3, and F4) in the topsoils (0–20 cm) and sediments in the two vineyards

Igeo	Zn	Pb	Ni	Ba	Mn	Cr	Cu
Tokaj–topsoil							
Mean	− 0.33	− 0.58	− 0.61	− 0.61	− 0.46	− 0.59	0.01
Min	− 0.81	− 0.76	− 0.77	− 0.97	− 0.54	− 0.81	− 0.86
Max	0.82	− 0.44	− 0.46	− 0.26	− 0.33	− 0.42	0.50
Tokaj–sediment							
Mean	0.32	− 0.17	− 0.28	− 0.18	− 0.44	− 0.25	1.92
Min	0.23	− 0.26	− 0.38	− 0.32	− 0.65	− 0.29	1.84
Max	0.47	− 0.11	− 0.13	− 0.05	− 0.12	− 0.19	2.03
Tállya–topsoil							
Mean	0.06	− 0.59	− 0.27	− 0.59	− 0.85	− 0.14	2.89
Min	− 0.38	− 0.88	− 1.20	− 1.28	− 1.43	− 1.13	1.94
Max	0.29	− 0.42	0.27	− 0.19	− 0.48	0.45	3.34
Tállya–sediment							
Mean	0.18	− 0.63	− 0.16	− 0.45	− 0.93	0.06	2.91
Min	− 0.04	− 1.07	− 0.32	− 0.72	− 1.49	− 0.18	2.75
Max	0.34	− 0.21	0.06	− 0.17	− 0.62	0.35	3.17

Nickel demonstrated relatively low extractability, with the residual fraction containing at least 73% of total Ni in Tokaj and 64% in Tállya, consistent with earlier research (Davidson et al., 2006; Lu et al., 2007). The reducible Ni fraction was comparable between sites, varying from 8–22% in Tállya and 11–17% in Tokaj. The significant positive correlations between Ni in the reducible (F2) and oxidizable (F3) fractions and silt content (Table 3) illustrate its association with fine soil particles, consistent with the high specific surface area and reactive sites of phyllosilicates and Fe/Mn oxyhydroxide coatings on silt particles (Kabata-Pendias, 2010). The positive correlations between Ni in reducible (F2: ρ = 0.70**) and residual (F4: ρ = 0.72**) fractions with soil pH and carbonate content (ρ = 0.73** and 0.82**, respectively) indicate favored Fe/Mn oxyhydroxide adsorption and incorporation into resistant mineral phases, such as phyllosilicates and carbonates, at higher pH (Roberts et al., 2005). This pH-driven immobilization mechanism is particularly evident in the alkaline Tokaj soils (pH 8.16 ± 0.07) compared to the slightly acidic Tállya soils (pH 6.38 ± 0.47), where Ni would be expected to exhibit greater mobility. The latter is also supported by Ni showing the strongest association with organic matter, reaching 19% in Tállya and 8% in Tokaj of oxidizable Ni. The calculated RAC consistently below 10% for Ni indicates no significant environmental risk in the studied vineyards.

In Tállya, the high proportion of extractable Cu (> 57%) down to a depth of 40 cm indicates clear anthropogenic contamination (El Azzi et al., 2013). Sequential extraction revealed that Cu contents were primarily residual (63%) in Tokaj and reducible (44%) in Tállya (Fig. 2). The gradually increasing ratio of extractable Cu toward surface layers in Tokaj’s soils also points to Cu accumulation from pesticide applications (Droz et al., 2021). These findings are consistent with the I_geo_ values indicating low to moderate contamination for Cu (0 < Igeo ≤ 1) in Tokaj topsoil (Table 4) and moderate to moderate-heavy contamination (1 < I_geo_ ≤ 3) in Tállya’s soils and sediments. Although Cu has a strong affinity for organic matter (Duplay et al., 2014), the oxidizable fraction remained minimal (≤ 5%) in both soils and sediments. Nevertheless, Cu-organic matter binding may still occur through readily soluble complexes extracted in the acid-soluble F1 fraction (Arenas-Lago et al., 2014), potentially accounting for up to 18% in Tállya. The consistent positive correlation with SOM across F1 (0.60**), F2 (0.68**), F3 (0.60**), and F4 (0.67**) (Table 3), may reflect co-variation with depth rather than a direct mechanistic relationship. The reducible fraction dominated in Tállya profiles, with an upward increasing trend in surface layers (from 28 to 48% in profile D). Similarly, reducible Cu was the most significant non-residual fraction in Tokaj (17–37%). Copper binding to Fe and Mn oxides and clay minerals is commonly observed across different soil types (Cerqueira et al., 2011; Vega et al., 2010). The elevated I_geo_ values (> 3 in some samples) at Tállya result from greater overall Cu enrichment and increased occurrence of labile Cu in soils due to slightly acidic soil pH results in medium risk level (RAC: 10–30%) (Elbana & Selim, 2011). In contrast, alkaline conditions and moderate Cu contamination at Tokaj-Hétszőlő highlighted by the lower contamination levels (mean I_geo_ of 0.01) result in lower environmental risk of Cu in the topsoil (RAC < 10%). Neaman et al. (2024) observed that acidic soils and high Cu mobility in traditional vineyards create contamination hotspots that extend to surrounding areas, especially during heavy rainfall. The present study demonstrates this wash-off of labile anthropogenic Cu through elevated sediment I_geo_ values, exceeding 2.91 at Tállya and surpassing 1.92 at Tokaj. These findings highlight the significance of contamination dispersal from vineyards to downslope environments.

XRPD analysis in Tokaj showed that topsoil consisted primarily of quartz (60–70%) with only 10–20% illite/muscovite, whereas eroded sediments contained larger amounts of illite/muscovite displaying 20–30% in H2 and 40–50% in H5 sediments. The high illite/muscovite content in sediments suggests elevated PTE transport within the sloping vineyards. SM&T data show that in Tokaj, all examined PTEs were more abundant in the acid-soluble and reducible fractions of eroded sediment compared to topsoil (Fig. 2). The highest mobility of Cu was also observed in Tokaj’s eroded sediments. Conversely, PTE fractionation in Tállya showed no marked differences between topsoil and eroded sediment. This similarity can be attributed to comparable mineral compositions in both materials (Babcsányi et al., 2016; Imfeld et al., 2020). So far, at the state of current Cu accumulation levels, sediments in Tokaj and topsoils in Tállya already reach the medium risk level (RAC: 10–30%). While Cu is an essential micronutrient for plant growth and soil microbial activity its increased soil-bound contents can impair soil health—defined as the capacity of soil to function as a living ecosystem supporting plant productivity, nutrient cycling, and microbial diversity—by inhibiting microbial communities, reducing enzymatic activities, and causing phytotoxicity in sensitive crops (Kabata-Pendias, 2010; Ruyters et al., 2013). Although the elevated Cu contents observed in this study (131 ± 38 mg/kg in Tállya, 51 ± 15 mg/kg in Tokaj) may approach phytotoxicity thresholds, particularly when present in mobile fractions, previous studies observed adverse effects attributed to soil-bound Cu typically above 200 mg/kg (Ruyters et al., 2013).

For Zn, the non-residual fraction constituted less than 20% in both Tokaj and Tállya (Fig. 2), suggesting relatively restricted extractability and indicating that most soil-bound Zn is integrated within mineral structures and likely of predominantly natural origin. However, Zn showed some anthropogenic accumulation in Tokaj, with maximum I_geo_ values reaching 0.82 in topsoil and even higher values in eroded sediments indicating their moderately contaminated status. In Tállya, I_geo_ values for both soils and sediments fell within the first two classes (I_geo_ ≤ 0 and 0 < I_geo_ ≤ 1), indicating uncontaminated to moderately contaminated status. At both vineyards, reducible Zn (F2) constituted the dominant extractable fraction, reflecting association with Fe/Mn oxyhydroxides (Nogueirol et al., 2010). The strong positive correlations between SOM and both the acid-soluble (F1: ρ = 0.73**) and reducible (F2: ρ = 0.65**) fractions of Zn (Table 3) suggest that organic matter promotes the retention of Zn in more mobile and bioavailable forms in accord with previous findings (Pérez-Rodríguez et al., 2023) and/or imply the co-variation of Zn and SOM with soil depth. Conversely, pH and carbonate content showed strong positive correlations with the residual fraction (F4: ρ = 0.66** and 0.77**, respectively), indicating that alkaline conditions promote Zn immobilization through incorporation into resistant mineral phases or carbonate precipitation. The RAC values showed contrasting patterns between the two sites. In Tállya, RAC values for all studied PTEs exhibited minimal variation between soils and sediments. In Tokaj, however, Zn displayed an inverse trend compared to other PTEs: RAC values were higher in topsoil (17.1%) than in sediment (13.0%), both falling within the medium risk category (RAC 10–30%).

Sequential extraction results for Ba revealed its predominant presence in the residual fraction (≥ 53% in Tokaj and ≥ 48% in Tállya) and reducible fraction (28% in both locations) (Fig. 2). The moderate Ba contents in the non-residual fractions indicate limited mobility, likely stemming from precipitation with sulfate and carbonate, as well as strong adsorption to clay minerals and Fe/Mn oxyhydroxides (Cappuyns, 2018; Sungur et al., 2019). The positive correlation between acid-soluble Ba (F1) and both pH (ρ = 0.41*) and CaCO_3_ (ρ = 0.50*) suggests that Ba is bound to carbonate fractions and stabilize in alkaline environments. Hence, Ba mobility could increase under acidic conditions, enhancing dissolution of carbonate-bound Ba and desorption from mineral surfaces. The positive correlation between residual Ba (F4) and carbonate content (ρ = 0.48*) reflects the incorporation of Ba into carbonate mineral structures. Despite its natural occurrence and low enrichment supported by I_geo_ values ≤ 0 in both soils and sediments at both sites, Ba exhibited medium risk levels (RAC: 10–30%) in Tokaj and low to medium risk in Tállya, indicating moderate bioavailability and toxicity risk.

### Practical implications for sustainable vineyard management

The findings of this study have direct implications for reducing PTE accumulation and preventing their transport to adjacent streams in wine-growing areas. Based on our sequential extraction results and environmental risk assessments, we recommend the following management strategies for winegrowers in historical wine-growing regions:

First, reducing Cu-based agrochemical input should be prioritized by winegrowers in medium to longer terms depending on the contamination status of their vineyards. Copper showed moderate to heavy contamination levels in the studied vineyards, reaching and in some cases exceeding the medium risk level (RAC ≥ 10%) in topsoils, with further enrichment observed in eroded sediments in Tokaj. Copper reduction strategy could include adopting integrated pest management approaches, utilizing alternative fungicides where feasible, and strictly adhering to minimum effective applicati Even though on rates. After decades of Cu application (e.g., 26 years in our study site at Tokaj and presumably even longer at Tállya), regular monitoring of mobile (F1) and bioavailable Cu fractions rather than total Cu contents provide a more realistic assessment of environmental risk. A periodic (every 3–5 years) assessment of mobile PTE fractions in both soils and any collected sediments is highly recommended, which can be the core of an effective early warning strategy for detecting increased PTE mobility and bioavailability.

Second, erosion control measures should be more effectively deployed in vineyards. Our results demonstrate significant PTE enrichment in eroded sediments, particularly for Cu (2.5–3.5 × higher than topsoils). To prevent PTE transport to streams draining the runoff from vineyards, winegrowers should construct sediment retention basins at slope bases (Babcsányi et al., 2014), leave vegetative buffer strips along waterways (Prosser et al., 2020), prefer contour farming on steep slopes, and use cover cropping between vine rows (Zuazo & Pleguezuelo, 2008). These measures are especially critical in vineyards planted on steep slopes and located in the proximity to aquatic ecosystems. In this study, Tállya vineyard, with its 18° mean slope and 10 m distance between the lower edge of the plot and the Vár stream, could benefit from the adoption of suggested erosion-control measures instead of tillage to better comply with sustainable management principles. In addition, the elevated Cu contents in the acid-soluble fraction highlight the risk of enhanced bioavailability and potential crop toxicity under the slightly acidic conditions in Tállya soils. Regular soil pH monitoring and lime applications if needed can help maintain PTEs in less mobile forms and reduce their environmental and agronomic risks.

These practices can help vineyard operators balance productive agriculture with protection of downstream aquatic ecosystems and long-term soil health.

## Conclusions

Overall, the residual fraction of PTEs predominated in the vineyard soils, with exceptions for Mn, Pb, and Cu in Tállya, and Pb and Mn in Tokaj, where non-residual fractions constituted the majority. Among the non-residual PTEs, the reducible fraction (PTEs associated with Fe and Mn oxyhydroxides) was the most abundant for the majority of PTEs examined here, except for Cr at both sites. In contrast, organically bound PTEs represented generally small proportions (except for Ni), despite the typically high metal affinity and sorption capacity of soil organic matter. The low organically bound fractions (F3) likely stem from the small SOM contents (< 2%) resulting from intensive erosion in these sloping vineyards.

The correlation analysis (Table 3) revealed that soil properties exert strong control over PTE fractionation patterns. Soil pH and carbonate content were major factors governing metal immobilization in the studied vineyards, as most PTEs showed positive correlations between the residual fractions and pH and carbonate content (e.g., Zn F4: ρ = 0.66** and 0.77**; Ni F4: ρ = 0.72** and 0.82**, respectively). However, it is important to note that strong pH-related correlations may partly reflect the binary site contrast rather than a mechanistic control of pH over PTE fractionation. Clay and silt content positively correlated with reducible fractions (F2) for several elements (Pb F2: ρ = 0.52**; Mn F2: ρ = 0.50**, respectively), indicating association of these elements with Fe/Mn oxyhydroxides coating fine-sized soil particles. Copper and Zn were minimally recovered in the oxidizable fraction (F3), generally accounting for the organically bound metal forms, SOM contents showed consistent positive correlations with Cu across all fractions (F1-F4: ρ = 0.60**− 0.68**) and with labile Zn fractions (F1-F2: ρ = 0.73** and 0.65**), which can rather be attributed to the co-variation of Cu/Zn and SOM in the vineyard soils (i.e., both decreasing with depth).

In Tállya, PTE fractionation patterns showed no notable differences between vineyard topsoil and eroded sediment. Conversely, Tokaj exhibited noticeable variations in both the enrichment levels and the geochemical fraction patterns of Zn, Pb, Ni, Ba, Mn, and Cu between soils and sediments. For Ba, Mn, and Cu, the most striking differences were observed in the acid-soluble and reducible fractions, showing higher percentages in sediments compared to topsoils, indicating substantially enhanced lability of these elements in transported materials. These variations are primarily attributable to differences in the particle size distribution and mineralogical composition, as sediments contained substantially higher illite/muscovite content (> 20%) compared to topsoils (10–20%), likely carrying higher proportions of PTEs bound to clay minerals and associated Fe and Mn oxyhydroxides. This selective transport of fine-grained, PTE-enriched soil particles represents a critical pathway for contaminant dispersal from vineyards to downslope areas, especially aquatic environments.

Comparing contamination and environmental risk assessment data between the two vineyards revealed similar patterns. While Ni, Cr, Mn, Ba, and Pb showed consistently low contamination (I_geo_ ≤ 0) across both sites due to their predominantly geogenic origin, supported by strong immobilization, and no/low enrichment, Zn and Cu demonstrated significantly elevated contamination levels, reflecting their anthropogenic sources and enhanced mobility during erosion events. Zinc showed moderate contamination in Tokaj sediments (mean I_geo_ = 0.32), while Cu exhibited moderate to heavy contamination in Tállya (mean I_geo_ = 2.89–2.91) and moderate contamination in Tokaj sediments (mean I_geo_ = 1.92). Despite lower pseudo-total Cu contents in Tokaj compared to Tállya, both sites reached medium environmental risk levels (RAC ≥ 10%) due to the substantial proportions of Cu in the F1 mobile fraction, particularly in transported sediments. These findings underscore the importance of assessing mobile/bioavailable PTE fractions rather than (pseudo-)total concentrations for monitoring contamination and environmental risks in vineyards, and highlight the urgent need to control soil erosion and prevent sediment transport to adjacent aquatic ecosystems, particularly vulnerable to PTE contamination. This study has several limitations that should be acknowledged. The relatively small sample size (n = 6 per matrix per site), the single sampling campaign, and the absence of sediment pH and grain-size data may constrain the generalizability of the findings.

## Data Availability

The authors declare that the data supporting the findings of this study are available within the paper. Should any raw data files be needed in another format (e.g., data of the sequential extractions) they are available from the corresponding author.
